# Analytical method development for simultaneous estimation of kojic acid, ascorbic acid and niacinamide in cosmetics and its validation

**DOI:** 10.1186/s13065-026-01741-0

**Published:** 2026-02-21

**Authors:** Vishnu Venkatesan, Gayatri Sukumaran, R. Lakshmi Sundaram, Chetan Ashok, S. Devaraja Dravida Pandiyan, Srikanth Jeyabalan, Mahendran Sekar, Ling Shing Wong, Vetriselvan Subramaniyan

**Affiliations:** 1 Department of Pharmaceutical Chemistry, Faculty of Pharmacy, Sri Ramachandra Institute of Higher Education and Research (DU), Porur, Chennai, Tamil Nadu 600116 India; 2 Department of Pharmacology, Faculty of Pharmacy, Sri Ramachandra Institute of Higher Education and Research (DU), Porur, Chennai, Tamil Nadu 600116 India; 3 School of Pharmacy, Monash University Malaysia, Bandar Sunway, Subang Jaya, Selangor Malaysia; 4 Faculty of Health and Life Sciences, INTI International University, Putra Nilai, Nilai, Negeri Sembilan 71800 Malaysia; 5 Department of Biomedical Sciences, Sir Jeffrey Cheah Sunway Medical School, Faculty of Medical and Life Sciences, Sunway University, Selangor Darul Ehsan, 47500 Malaysia

**Keywords:** Kojic acid, Ascorbic acid, Niacinamide, Cosmetic cream, HPLC, Public health

## Abstract

A novel HPLC method was developed and validated for the concurrent measurement of kojic acid, ascorbic acid, and niacinamide in cosmetic formulations. Using methanol and 0.1% acetic acid gradient elution on a C18 column, the method enables efficient separation and quantification of all three active components within 15 min, markedly improving analytical speed and solvent economy compared to previous approaches. Rigorous method validation demonstrated outstanding precision, specificity, linearity (r2 > 0.99), and robustness were developed in accordance with ICH criteria with the system appropriateness factors, like the tailing factor and theoretical plate count, consistently meeting acceptance criteria. The limits of detection were as low as kojic acid (0.06 µg/mL), niacinamide (0.10 µg/mL), and ascorbic acid (0.15 µg/mL), while percent recoveries for all analytes ranged between 98% and 102% at multiple concentration levels, confirming accuracy. Repeatability and intermediate precision (%RSD < 2%) were upheld across replicate assays, underlining the method’s reliability for routine quality control. The unique advantage of this method lies in its simultaneous, rapid assessment of multi-functional vitamins and antioxidants common to cosmetics, streamlining regulatory compliance and product development. This robust protocol provides a practical solution for manufacturers and research laboratories seeking fast, precise, and economical analysis of active ingredients in commercial skincare products.

## Introduction

**Fig. 1:**
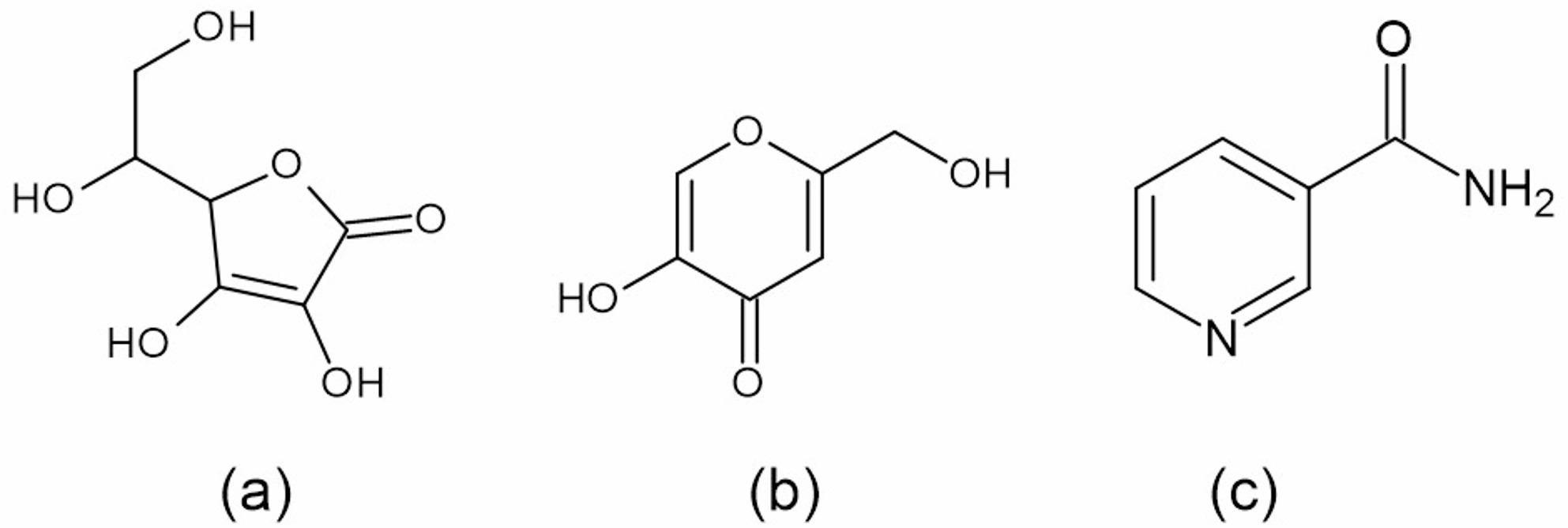
**a** Ascorbic acid **b** Kojic acid **c** Niacinamide

Kojic acid (5-hydroxymethyl-2-(hydroxymethyl)-4-pyrone) functions by inhibiting the conversion of tyrosine, a key step in melanin synthesis. By suppressing melanin formation, it can contribute to a skin-lightening effect. Molecular weight is 114.11 g/mol. It is used for acne scars, hyperpigmentation [1]. Ascorbic acid (5R)-[(1 S)-1,2-Dihydroxyethyl]2,5 H-one − 3,4-dihydroxyfuran serves as a potent antioxidant, cofactor, co-substrate, and enzyme complement in a variety of reactions and metabolic processes [2]. Niacinamide (pyridine-3-carboxamide) supports the formation of skin cells and provides protection against environmental factors including UV exposure, pollutants, and harmful toxins. It is used for severe acne, especially inflammatory forms like papules and pustules [3]. The structure of selected compounds is shown in Fig. 1.

The methods developed for kojic acid [4–8] and niacinamide [8–11] previously employed high-performance liquid chromatography (HPLC) and an ultraviolet visible spectrophotometer [4–6], Ascorbic acid [12, 13] was also quantified by different instruments. More recently, Permana et al. (2023) developed a simultaneous HPLC method for niacinamide and ascorbic acid derivatives in the presence of parabens [14]. Furthermore, advanced techniques such as UPLC-MS/MS have been employed by recent studies (2023) to quantify complex mixtures of whitening agents including nicotinamide and kojic acid, though these methods often require expensive instrumentation not available in all quality control settings [15]. Recent surveys of the cosmetic market (2024) continue to highlight the need for robust quantification of these active ingredients to ensure regulatory compliance [16].

To the best of our knowledge, no method has been reported for the simultaneous estimation of this specific combination of ingredients in a single chromatographic run. The objective of this work was to develop a novel, rapid, and economical HPLC method to quantify Kojic acid, Ascorbic acid, and Niacinamide simultaneously in commercial cosmetic formulations.

## Methodology

### Materials

Kojic acid (purity > 99.0%) was purchased from Crysti Chemicals, Mumbai. Reference standards of Niacinamide (purity 99.8%) and Ascorbic acid (purity 99.5%) were kindly provided by Caplin Point Laboratories, Chennai. Methanol was procured from Adevent (HPLC grade). All of the other reagents and solvents were analytical grade. The instruments Shimadzu P-series automatic sampler HPLC, electronic balance (Sartorius), and ultra sonicator (Ilexco) were used.

### HPLC operating conditions

Chromatographic separation and simultaneous analysis were carried out utilizing an HPLC system equipped with a PDA detector operated in gradient mode. A 250 mm × 4.6 mm, 5 μm C18 column; Waters) served as stationary phase. Instrument parameters were configured with a 1.0 mL/min flow rate, a 10 µL injection volume, and a 40 °C column temperature. The PDA detector was set to scan the range of 200–600 nm, and the chromatograms were extracted at a detection wavelength of 272 nm. Several mobile phase combinations were evaluated based on resolution, theoretical plates, capacity factor, and other system suitability parameters. The final optimized separation employed a 0.1% acetic acid (channel B) and methanol (channel A) gradient elution, 5% B at 0.01 min and 25% B at 15 min is the gradient’s preset progression.

#### Method optimization

During the development phase, various chromatographic conditions were trialed to achieve optimal resolution and peak symmetry. Initial trials using isocratic elution with water and methanol (Trials 1 & 2) resulted in broad peaks and poor resolution between Niacinamide and Kojic acid.

The addition of 0.1% acetic acid (Trials 3 & 4) was found to significantly improve peak shape by suppressing ionization. However, isocratic conditions were insufficient for complete separation. Therefore, a Gradient mode was introduced in Trial 5 (5% to 25% organic phase), which resulted in sharp, well-resolved peaks. Flow rates were also optimized, and 1.0 mL/min was selected as it provided the best balance between run time and column backpressure. The summary of optimization trials is presented in Table 1.

### Preparation of stock and standard solutions

#### Kojic acid

About 100 mg of kojic acid reference standard was precisely weighed and transferred into a 100 mL volumetric flask. Methanol (100 mL) was added, stirred well, and sonicated for five minutes, and then 5 mL of solution was carefully pipetted using micropipette and moved into a 100 mL volumetric flask, and distilled water was used to adjust the volume. The concentration of the resulting stock solution was 50 µg/mL.

#### Ascorbic acid

About 100 mg of ascorbic acid reference after precisely weighing the standard, it was put into a 100 mL volumetric flask. After adding 100 mL of methanol and vigorously mixing it, it was sonicated for five minutes, and then 25 mL of solution was carefully pipetted using micropipette and moved into a 100 mL volumetric flask, and distilled water was added to adjust the volume. The concentration of the resulting stock solution was 250 µg/mL.

**Table 1:** Optimization of chromatographic conditions

Chromatographic Parameter	Trial 1	Trial 2	Trial 3	Trial 4	Trial 5
Mobile Phase	Methanol: water – 40: 60 (%)	Methanol: water – 10: 90 (%)	Methanol (0.1% acetic acid): water – 10: 90 (%)	Methanol (0.1% acetic acid): water – 05: 95(%)	Methanol (0.1% acetic acid): water – 10: 90 (%)
Column selected	Waters C18 250 * 4, 60 mm 5 micron	Waters C18 250 * 4, 60 mm 5 micron	Waters C18 250 * 4, 60 mm 5 micron	Waters C18 250 * 4, 60 mm 5 micron	Waters C18 250 * 4, 60 mm 5 micron
Mode	Isocratic mode	Isocratic mode	Isocratic mode	Isocratic mode	Gradient mode 5% − 25% (0.01–15 min)
Column temperature	40 °C	40 °C	40 °C	40 °C	40 °C
Run time	10 min	10 min	15 min	15 min	15 min
Injection volume	10 µl	10 µl	10 µl	10 µl	10 µl
UV wavelength	Kojic acid at 269 nm, Ascorbic acid at 257 nm, niacinamide at 262 nm	Kojic acid at 269 nm, ascorbic acid at 257 nm, niacinamide at 262 nm	Kojic acid at 269 nm, ascorbic acid at 257 nm, niacinamide at 262 nm	Kojic acid at 269 nm, ascorbic acid at 257 nm, niacinamide at 262 nm	Kojic acid at 269 nm, ascorbic acid at 257 nm, niacinamide at 262 nm
Observation	Broad peaks; poor resolution.	Poor resolution between analytes.	Improved peak shape but incomplete separation.	Better separation but long run time.	Sharp peaks; Resolution > 2.0. (Final Method)

#### Niacinamide

About 100 mg of niacinamide reference after being precisely weighed, the standard was put into a 100 mL volumetric flask. After adding 100 mL of methanol, it was well combined and sonicated for five minutes, and then 25 mL of solution was carefully pipetted using micropipette and moved into a 100 mL volumetric flask, and distilled water was used to adjust the volume. The concentration of the resulting stock solution was 250 µg/mL.

### Preparation of marketed cosmetic formulation

To assess the method’s applicability, a commercial cosmetic product, Deconstruct Niacinamide Brightening Face Moisturizer, manufactured by Intigree Biomed Private Limited (Mumbai, India), labeled to contain Kojic acid (1% w/w), Niacinamide (5% w/w), and Ascorbic acid (5% w/w), was selected for analysis.

#### Extraction procedure

Five hundred milligrams (500 mg) of the sample cream was accurately weighed and transferred into a centrifuge tube. Ten milliliters (10 mL) of methanol was added, and the mixture was centrifuged at 4000 rpm for 30 min. The supernatant was collected, and a 1.0 mL aliquot was transferred into a 10 mL volumetric flask. The volume was made up to the mark with methanol, and the solution was filtered through a 0.45 μm PTFE filter. This extraction procedure was repeated six times (*n* = 6), and the resulting solutions were injected into the HPLC system.

#### Placebo analysis

A placebo mixture consisting of the cream base without the active ingredients was prepared and treated in the same manner (extraction and filtration) to verify the absence of interference at the retention times of the analytes.

### Specificity

The capacity to evaluate analyte presence uniformly across potential constituents is known as specificity. By contrasting the peak area of the sample with that of the standard solution, the specificity was ascertained. Any interference from the blank was examined in the sample.

### Precision

The degree of agreement (or scatter) between a set of measurements made by repeatedly sampling the same homogenous sample under specified conditions is expressed as the precision of the analytical process. Repeatability, intraday precision, and interday precision were used to calculate precision.

### Linearity

Standard stock solution aliquots were diluted to concentrations ranging from 100 µg/mL to 3.125 µg/mL for kojic acid and 500 µg/mL to 15.625 µg/mL for ascorbic acid and niacinamide. Peak area versus concentration for each of the three compounds was used to create the calibration curve.

### Repeatability

Analyzing six samples with the same medication concentration allows for the measurement of repeatability. Each chromatogram’s area was reported after it was recorded.

### Robustness

The method’s ability to hold steady in the face of slight conditional changes is what makes it resilient. To determine the assay’s robustness, the flow rate and temperature were altered.

### Recovery

Recovery tests were conducted using the standard at 80%, 100%, and 120% levels for previously analyzed samples (10 µg/mL) and subsequent samples were reanalyzed in order to guarantee method accuracy. Three determinants were carried out at each stage. Accuracy is expressed as a percentage of recovery, and the formula to compute it is.

(Observed value * 100) / True value = % Recovery [5].

### Solution stability

To assess the stability of the analytes, standard and sample solutions were stored at room temperature (25 ± 2 °C) and analyzed at intervals of 0, 12, and 24 h. The peak areas obtained at the initial time point were compared with those obtained at subsequent intervals. The percentage relative standard deviation (%RSD) for the peak areas was calculated to determine the stability of Kojic acid, Niacinamide, and Ascorbic acid in the mobile phase [17].

## Results and discussion

**Fig. 2:**
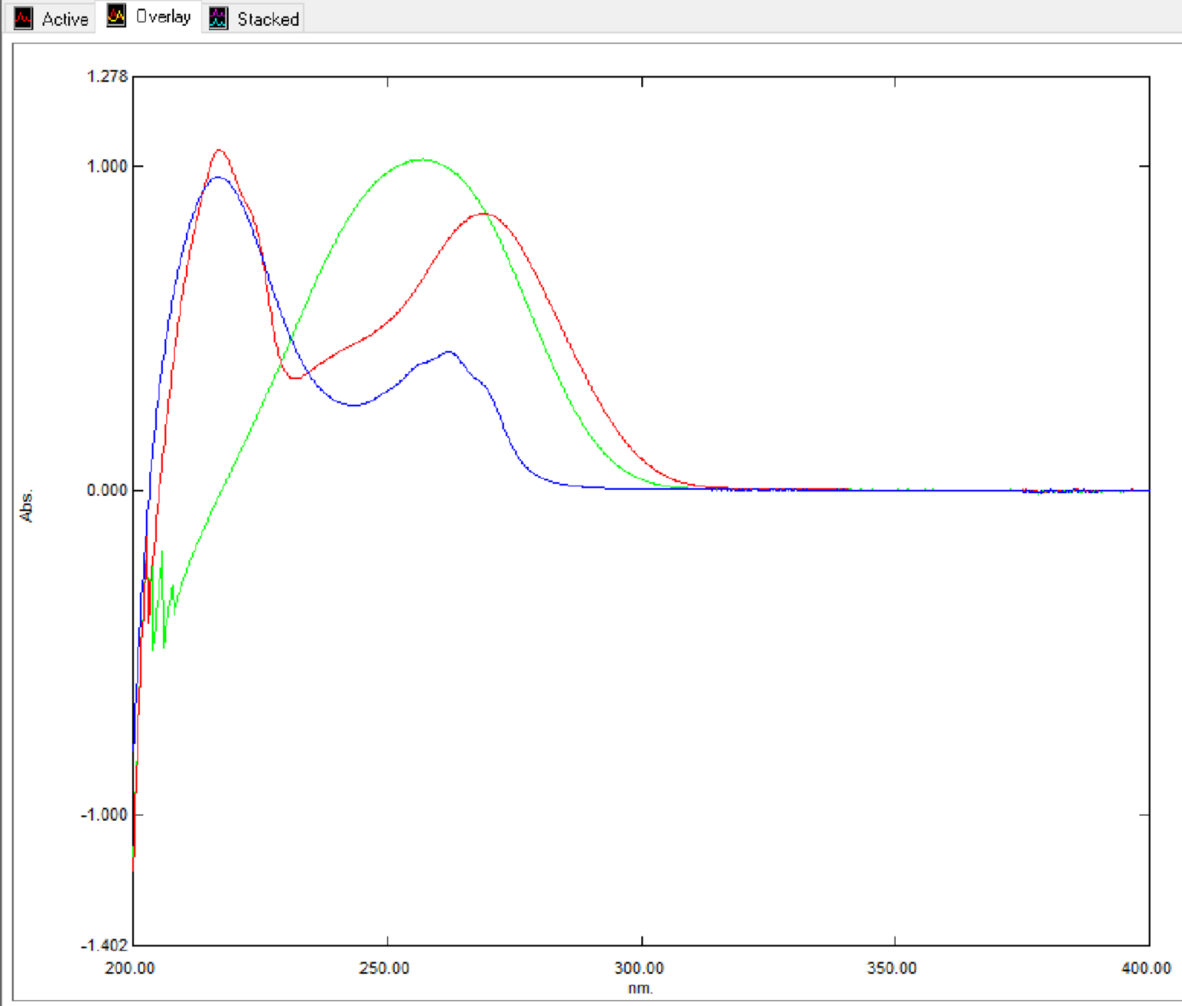
Overlay UV absorption spectra of the three analytes scanned between 200–400 nm: Kojic acid (Red line) showing λ_max_ at 269 nm; Ascorbic acid (Green line) showing λ_max_ at 257 nm; and Niacinamide (Blue line) showing λ_max_ at 262 nm

Standard kojic acid was found to have two λ_max_ at 269 and 217 nm. Standard ascorbic acid was found to have λ_max_ at 257 nm. Standard niacinamide was found to have two λ_max_ at 262 and 216 respectively (Fig. 2).

**Fig. 3:**
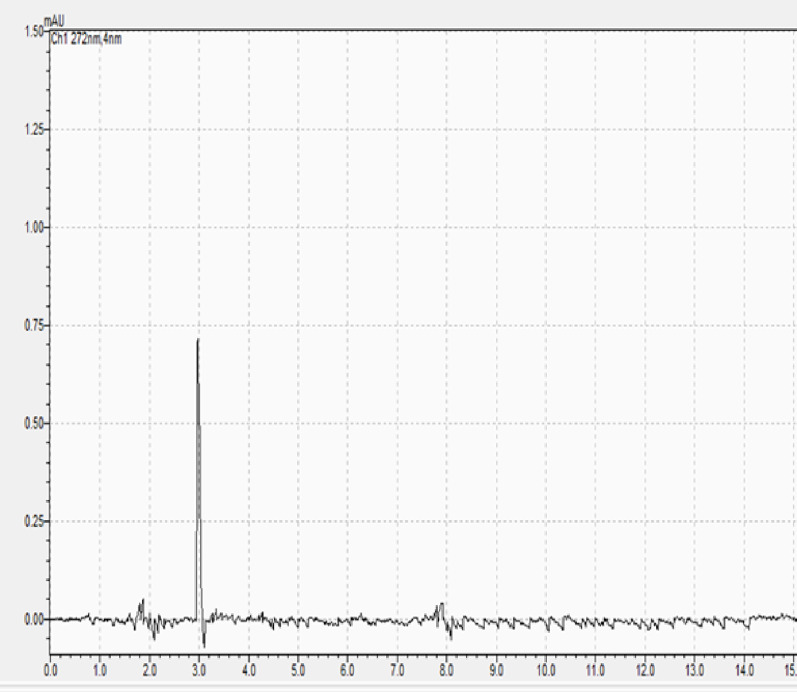
Chromatogram of the placebo formulation. The peak observed at 3.0 min corresponds to the solvent front/void volume. No interfering peaks were detected at the retention times of the analytes

### System suitability

The suitability of the system was assessed by injecting a standard concentration of 250 µg/mL of niacinamide and ascorbic acid, and 50 µg/mL of kojic acid into HPLC system and the peak area, tailing factor and resolution were obtained and presented in Table 2. The number of theoretical plates for all the three peaks were above 2000 and tailing factor of all the three peaks were less than 2%.

**Table 2:** System suitability parameters for Kojic acid

Parameter	Kojic acid	Niacinamide	Ascorbic acid	Acceptance criteria
Number of theoretical plates	4613	10,891	15,616	≥ 2000
Tailing factor	1.619	1.039	1.884	≤ 2
Resolution		3.678	11.709	≥ 3

### Specificity

The specificity of the method was established by injecting a blank, a placebo formulation (cream base without actives), and the standard solution. As shown in Fig. 3 (Placebo Chromatogram), no interfering peaks were observed from the excipients at the retention times of Kojic acid, Niacinamide, or Ascorbic acid. Peak purity shows that at the retention time of the corresponding pharmaceuticals; all three peaks were homogenous and free of co-eluting peaks. The ICH standards are met by all three peaks (Table 3).

**Table 3:** Compound and their retention time for specificity

Compound	Retention time (mins)
Acetic acid (used in mobile phase)	2.9
Kojic acid	6.5
Niacinamide	7.8
Ascorbic acid	11.7

### Precision

**Fig. 4:**
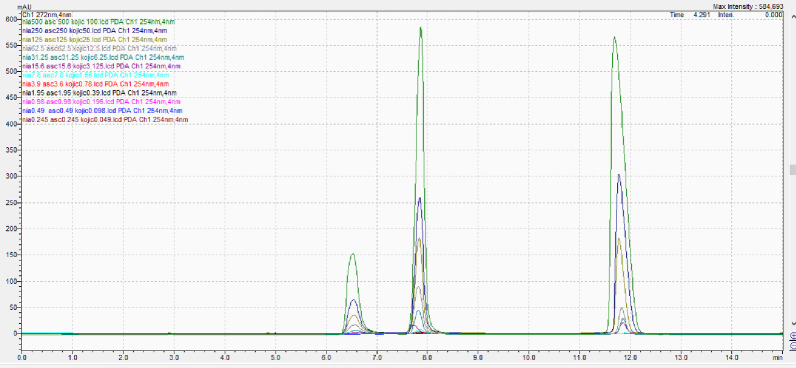
Chromatogram of standards kojic acid, niacinamide and ascorbic acid for precision eluted at 6.5, 7.8 and 11.7 min respectively

The API standard was prepared for concentration of 250 µg/mL of niacinamide and 50 µg/mL of kojic acid and ascorbic acid. The above preparation was prepared six times and added to the HPLC apparatus, and the peak region of all the peaks were noted and the % RSD was calculated. The % RSD values for all compounds were found to be less than 2% (Table 4; Fig. 4), which meets the acceptance criteria specified by the International Council for Harmonisation Q2(R1) guideline for precision, indicating the method is reproducible and precise under the prescribed conditions.

**Table 4:** Peak area of Kojic acid, niacinamide and ascorbic acid

S No	Peak area of kojic acid	Peak area of niacinamide	Peak area of ascorbic acid	Acceptance criteria
1.	1,050,969	2,923,198	3,956,590	≤ 2
2.	1,081,342	3,002,346	4,136,729	
3.	1,072,657	2,986,830	4,100,834	
4.	1,068,626	2,975,436	4,078,435	
5.	1,065,882	2,980,128	3,979,657	
6.	1,066,006	2,978,662	3,968,973	
Average	1067580.3	2,974,433	4086869.66	
% RSD	0.934	0.904	0.629	

### Linearity

**Fig. 5:**
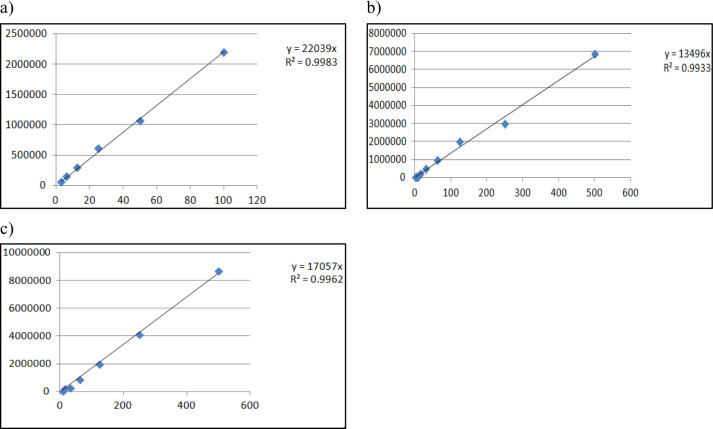
Linearity graph for **a**) Kojic acid **b**) Niacinamide **c**) Ascorbic acid

Different concentration of API ranging from 500 µg/mL to 31.25 µg/mL for niacinamide and ascorbic acid and from 100 µg/mL to 7.8 µg/mL for kojic acid were prepared and injected into HPLC system and after building a calibration curve between concentration and absorbance, the r2 value was determined to be larger than 0.99 for all three compounds which is in accordance with ICH guidelines (Fig. 5).

### LOD & LOQ

The lowest amount of an analyte that can be detected but not necessarily quantified is known as the limit of detection (LOD) (Table 5), whereas the lowest amount that can be quantitatively identified with appropriate precision is known as the limit of quantification (LOQ) [18]. The LOD and LOQ were calculated using the standard deviation of response (σ) and the slope (*S*) of the calibration curve according to the equations, LOD = 3.3σ/*S* and LOQ = 10σ/*S*, where σ is the standard deviation of the y-intercepts of the regression lines and *S* is the slope of the calibration curve.

**Table 5:** LOD and LOQ for Kojic acid, niacinamide and ascorbic acid

S No	Name of the drug	LOD (µg/mL)	LOQ (µg/mL)
1.	Kojic acid	0.06	0.21
2.	Niacinamide	0.1	0.3
3.	Ascorbic acid	0.15	0.45

### Robustness

Flow rate and column temperature was changed to verify the developed method’s resilience. Flow rate changed at maximum value of 1.3 mL and minimum value of 0.7 mL and column temperature was changed as maximum value of 43 °C and minimum value of 37 °C there was no change in peak area with change in flow rate as well as column temperature and the developed method is robust according to ICH guidelines (Table 6).

**Table 6:** Robustness

Compound name	Flow rate	Peak area	Column temperature	Peak area
Kojic acid	1 mL	1,056,457	40 °C	1,060,943
	1.3 mL	1,048,235	43 °C	1,048,521
	0.7 mL	1,035,754	37 °C	1,059,024
Niacinamide	1 mL	2,952,346	40 °C	3,002,346
	1.3 mL	2,845,734	43 °C	2,986,830
	0.7 mL	2,834,583	37° C	2,975,436
Ascorbic acid	1 mL	3,974,463	40 °C	3,985,442
	1.3 mL	3,953,452	43 °C	4,036,729
	0.7 mL	3,946,234	37 °C	4,020,834

### Assay

**Fig. 6:**
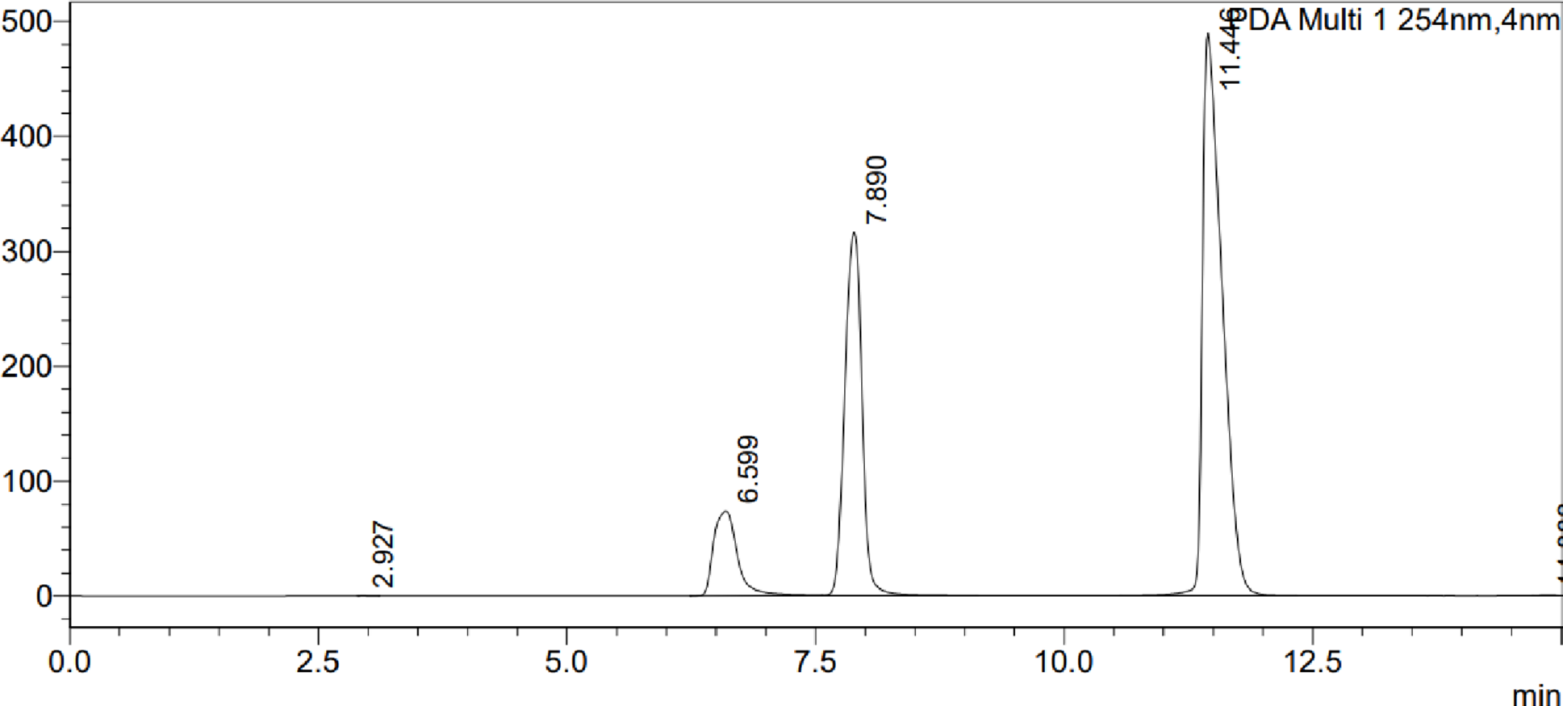
Typical HPLC chromatogram of the standard mixture. The chromatogram displays symmetric peak shapes and baseline resolution for (1) Kojic acid (t_R_ 6.59 min), (2) Niacinamide (t_R_ 7.89 min), and (3) Ascorbic acid (t_R_ 11.44 min). The peak at 2.92 min corresponds to the solvent front

Sample was prepared six times at the concentration of 50 µg/mL of kojic acid, 250 µg/mL of niacinamide and 250 µg/mL of ascorbic acid and injected into HPLC system and the chromatogram was recorded (Fig. 6). The amount found was calculated and the % RSD was calculated for all six injections for all three compounds and it was within 2 and it is according to ICH guidelines (Table 7).

**Table 7:** Assay of Kojic acid, niacinamide and ascorbic acid

	Kojic acid		Niacinamide		Ascorbic acid	
	Concentration	Amount found	Concentration	Amount found	Concentration	Amount found
% RSD	50	50.23	250	249.91	250	249.53
		49.82		249.25		250.51
		50.45		250.52		249.62
		50.37		249.62		248.92
		48.94		250.21		250.02
		50.71		249.74		248.42
	0.934		0.903		1.02	

### Recovery

The recovery was determined by preparing three different concentration (mixture of standard and sample) 80%, 100%, 120%. The results of recovery for all three compounds were expressed as percentage recovery and it was found to be within the range of 98–102% and the % RSD was within 2% and it satisfies the ICH guidelines (Table 8).

**Table 8:** Recovery for Kojic acid, niacinamide and ascorbic acid

Level	Kojic acid			Niacinamide			Ascorbic acid		
	Concentration	% Recovery	% RSD	Concentration	% Recovery	% RSD	Concentration	% Recovery	% RSD
80	40	99.80	0.626	225	99.69	0.732	225	100.28	0.739
	40	98.52		225	98.88		225	99.21	
	40	99.30		225	100.03		225	99.72	
100	50	100.1	0.377	250	99.83	0.844	250	99.52	0.824
	50	99.70		250	100.24		250	99.90	
	50	99.84		250	99.49		250	99.24	
120	60	99.90	0.639	275	100.07	0.911	275	99.97	0.612
	60	100.5		275	99.44		275	100.07	
	60	99.76		275	99.68		275	99.61	

### Solution stability

The stability of the analytical solutions was assessed by analyzing the standard and sample preparations at intervals of 0, 12, 24, 36, and 48 h. The peak areas were recorded, and the % RSD was calculated. As shown in Table 9, the % RSD for all three analytes was found to be less than 2%, indicating that the solutions remain stable for up to 48 h at room temperature.

**Table 9:** Solution stability data for Kojic acid, Niacinamide, and ascorbic acid (*n* = 6)

Time Interval	Kojic Acid (Peak Area)	Niacinamide (Peak Area)	Ascorbic Acid (Peak Area)
Initial	1,050,969	2,923,198	3,834,724
After 12 h	1,068,242	2,845,724	3,938,217
After 24 h	1,063,451	2,834,547	3,952,754
After 36 h	1,047,542	2,754,764	3,782,421
After 48 h	1,057,426	2,676,425	3,875,242
Average	1,057,526	2,846,931	3,876,671
% RSD	0.81%	0.71%	1.23%

## Conclusion

In this study, a novel HPLC-PDA method was successfully developed and validated for the simultaneous estimation of Kojic acid, Niacinamide, and Ascorbic acid in cosmetic formulations. To the best of our knowledge, this is the first reported method capable of quantifying this specific combination of skin-whitening agents in a single chromatographic run. Unlike previous methods that relied on expensive and less eco-friendly solvents like acetonitrile, the present method utilizes a cost-effective methanol and 0.1% acetic acid mobile phase. The method demonstrated excellent precision, accuracy, and robustness, with a rapid run time of fewer than 15 min. These attributes make it highly suitable for routine quality control in the pharmaceutical and cosmetic industries, ensuring regulatory compliance and consumer safety.

## Limitation

One of the study’s limitations is that the HPLC method that was developed was validated only using standard solutions and a specific cosmetic formulation, without testing its applicability across diverse and complex cosmetic products. Additionally, the method employs UV detection, which may limit sensitivity for detecting degradation products or impurities. Future work should explore application of this method to various formulations and incorporate more sensitive detectors like mass spectrometry for comprehensive analysis, including impurities and stability studies, thereby enhancing method robustness and broader utility in cosmetic quality control.

## Data Availability

The datasets used and/or analysed during the current study are available from the corresponding author on reasonable request.

## Abbreviations

API: Active Pharmaceutical Ingredient
PDA: Photodiode Array detector
HPLC: High-Performance Liquid Chromatography
RSD: Relative Standard Deviation (commonly written as %RSD)
LOQ: Limit of Quantification
ICH: International Council for Harmonisation of Technical Requirements for Pharmaceuticals for Human Use
LOD: Limit of Detection
